# Diagnostic accuracy of the postoperative ratio of C-reactive protein to albumin for complications after colorectal surgery

**DOI:** 10.1186/s12957-016-1092-1

**Published:** 2017-01-10

**Authors:** Xiaolong Ge, Yu Cao, Hongkan Wang, Chao Ding, Hongliang Tian, Xueying Zhang, Jianfeng Gong, Weiming Zhu, Ning Li

**Affiliations:** 1 Department of General Surgery, Jinling Hospital, Medical School of Nanjing University, 305 East Zhongshan Road, Nanjing, 210002 China; 2 Drum Tower Hospital, Medical School of Nanjing University, Nanjing, China; 3 First Affiliated Hospital, Zhejiang University School of Medicine, Hangzhou, China

**Keywords:** C-reactive protein to albumin ratio, Colorectal surgery, Diagnoses

## Abstract

**Background:**

The ratio of C-reactive protein to albumin, as a novel inflammation-based prognostic score, is associated with outcomes in cancer and septic patients. The diagnostic accuracy of the CRP/albumin ratio has not been assessed in colorectal surgery for postoperative complications.

**Methods:**

A total of 359 patients undergoing major colorectal surgery between 2012 and 2015 were eligible for this study. Uni- and multivariate analyses were performed to identify risk factors for postoperative complications. Receiver operating characteristic curves were developed to examine the cutoff values and diagnostic accuracy of the CRP/albumin ratio and postoperative CRP levels.

**Results:**

Among all the patients, 139 (38.7%) were reported to have postoperative complications. The CRP/albumin ratio was an independent risk factor for complications (OR 4.413; 95% CI 2.463–7.906; *P* < 0.001), and the cutoff value was 2.2, which had a higher area under the curve compared to CRP on postoperative day 3 (AUC 0.779 vs 0.756). The CRP/albumin ratio also had a higher positive predictive value than CRP levels on postoperative day 3. Patients with CRP/albumin ≥2.2 suffered more postoperative complications (60.8% vs 18.6%, *P* < 0.001), longer postoperative stays (10 (4–71) vs 7 (3–78) days, *P* < 0.001), and increased surgical site infections (SSIs) (21.1% vs 4.8%, *P* < 0.001) than those with CRP/albumin <2.2.

**Conclusions:**

The ratio of C-reactive protein to albumin could help to identify patients who have a high probability of postoperative complications, and the ratio has higher diagnostic accuracy than C-reactive protein alone for postoperative complications in colorectal surgery.

## Background

There are various postoperative complications occurring after surgery for both benign and malignant colorectal disease [1, 2], and they cause increased treatment costs, infections, longer hospital stays, a slower return to premorbid status and worse long-term survival [3–5]. Therefore, successful early detection and treatment of any complications are paramount to improve outcomes, especially under the enhanced recovery after surgery (ERAS) program.

As surgical interventions lead to well-understood metabolic, neuroendocrine, and immune responses, the stress responses to surgery contribute to increased postoperative complications [6]. Pro-inflammatory cytokines will increase due to surgical injury, which leads to changes of circulating acute phase proteins, such as albumin and C-reactive protein (CRP) [6]. It is widely known that CRP is used to detect postoperative complications resulting from surgical injury. Additionally, several studies found CRP to be a useful tool to ensure a safe early discharge following colorectal surgery [7]. Warschkow et al. [1] reported that CRP on postoperative day 3 or 4 (POD 4) had the best performance in predicting postoperative complications. On the other hand, some studies have proven that low albumin levels are related to postoperative complications. Increased capillary leakage of albumin is one of the features of systemic inflammatory response syndrome (SIRS), which leads to low plasma albumin levels in patients undergoing major abdominal surgery [8]. A pilot study has reported that an early postoperative albumin drop was associated with adverse clinical outcomes and was due to surgical trauma [9]. Lee et al. [10] reported that patients with postoperative hypoalbuminemia were more likely to experience poorer outcomes undergoing off-pump coronary artery bypass graft surgery.

A recent study has proven that a novel inflammation-based prognostic score, the CRP/albumin ratio (CAR), is a useful predictive index for outcome in patients with sepsis or colorectal cancer [11, 12]. However, almost no study has investigated the diagnostic accuracy of CAR for postoperative complications in patients undergoing colorectal surgery. Therefore, we hypothesized that CAR could be used as a reliable and accurate predictor of postoperative outcome after colorectal surgery.

In the present study, we investigated the association between CAR and postoperative complications after colorectal surgery and compared the diagnostic accuracy between CAR and postoperative CRP in these patients.

## Methods

### Patients

This study was approved by the ethics committee of Jinling Hospital. The information on patients undergoing elective colectomy or rectal resection for malignant or non-malignant disease was retrospectively analyzed at the Department of General Surgery in Jinling Hospital between October 2012 and December 2015. Patients with reoperation prior to postoperative day 3, albumin infusion either preoperatively or within 2 postoperative days, closing of ileostomy or colostomy, multivisceral resection, inflammatory bowel disease, or incomplete laboratory data were excluded. Patients who underwent primary intestinal resection were generally included.

### Data collection

The data collected included baseline characteristics, intraoperative data and laboratory data from the database. Baseline characteristics included age, sex, body mass index, American Society of Anesthesiologists (ASA) grade, comorbidities, and surgical indication. Laboratory data included hemoglobin, preoperative CRP and albumin, CRP on POD 3 [7], and albumin on POD 3 [13]. Intraoperative data included operation time, type of surgery, surgical approach (open vs laparoscopy), stoma creation, intraoperative blood transfusion, and estimated blood loss during surgery.

### Definition of outcomes

Postoperative outcomes were evaluated, including days of postoperative hospital stay, surgical site complications (surgical site infections (SSIs)), and postoperative complications (Clavien-Dindo classification) [14]. The primary outcome was overall postoperative complications graded according to the Clavien-Dindo system. Grades I to II were defined as mild complications, and grades III to IV were defined as major complications. Postoperative complications were defined as those that occurred before hospital discharge or <30 days after surgery. Surgical site complications included superficial incisional, deep incisional, or organ/space SSIs. For evaluation, CAR was defined as (CRP level on POD 3)/(ALB level on POD 3) [1, 13, 15]. The CAR cutoff values were determined using receiver operating characteristic (ROC) curve analysis [11].

### Statistical analysis

All of the data were analyzed using SPSS version 19.0 (SPSS, Inc., Chicago, IL). Continuous data are presented as the mean ± SD or median (range), while categorical data are presented as *n* (%). Continuous variables were analyzed using Student’s *t* test, categorical variables were analyzed using Pearson’s chi-square test or the Fisher exact test as appropriate. Significant associations (*P* < 0.05) on univariate analysis were submitted to multivariate logistic regression analysis to verify independent predictors of postoperative complications. ROC curve analysis was used to assess the predictive accuracy. *P* values <0.05 were considered statistically significant.

## Results

### Study population and baseline characteristics

A total of 359 patients were enrolled for the final analysis (male to female = 179:180), of which 83.3% had malignant disease, as shown in Table 1. Among those with benign disease, 60 (16.7%) were chronic radiation enteropathy, diverticular disease or others. There were 139 (38.7%) patients who had postoperative complications. In addition, 96 (26.7%) patients had mild complications, and 55 (15.3%) patients had major complications (Clavien-Dindo grade III−IV), and 12.5% patients had SSIs. The length of the postoperative hospital stay was 11.2 ± 10.2 days. The clinical background characteristics of the two groups of studied patients with or without postoperative complications are shown in Table 1.

**Table 1:** Univariate analysis of risk factors associated with postoperative complications

Characteristics	All	Postoperative complications		*P* value
	(*n* = 359)	Yes (*n* = 139)	No (*n* = 220)	
Age (year)^a^	53.4 ± 10.4	53.1 ± 9.6	53.5 ± 10.8	0.717
Sex, male, *n* (%)^c^	179 (49.9)	52 (37.4)	127 (57.7)	<0.001
BMI (kg/m^2^)^a^	22.3 ± 3.7	21.6 ± 3.5	22.7 ± 3.7	0.004
Comorbidities^c^				
Diabetes mellitus	49 (13.6)	16 (11.5)	38 (17.3)	0.137
Hypertension	113 (31.5)	40 (28.8)	73 (33.2)	0.381
Preoperative hemoglobin (g/L)^a^	120.7 ± 58.6	109.4 ± 26.9	127.8 ± 70.9	0.004
Preoperative ALB (g/L)^a^	40.6 ± 3.8	40.2 ± 4.0	40.8 ± 3.7	0.185
Preoperative CRP (mg/L)^a^	9.5 ± 23.8	12.2 ± 27.1	7.9 ± 21.3	0.097
Operation time (min)^b^	160 (70–383)	160 (70–365)	165 (70–383)	0.483
Type of surgery, *n* (%)^c^				0.117
Right colectomy	126 (35.1)	60 (43.2)	66 (30.0)	
Transverse colectomy	14 (3.9)	5 (3.6)	9 (4.1)	
Left colectomy	44 (12.3)	19 (13.7)	25 (11.4)	
Sigmoid resection	41 (11.4)	12 (8.6)	29 (13.2)	
Rectal resection	116 (32.3)	38 (27.3)	78 (35.5)	
Total colectomy	18 (5.0)	5 (3.6)	13 (5.9)	
Laparoscopic surgery, *n* (%)^c^	142 (39.6)	49 (35.3)	93 (42.3)	0.185
Conversion, *n* (%)^c^	25 (7.0)	11 (7.9)	14 (6.4)	
Estimated blood loss (ml)^a^	226 ± 137	245 ± 139	214 ± 134	0.035
Stoma creation, *n* (%)^c^	58 (16.2)	30 (21.6)	28 (12.7)	0.026
CRP on POD 3 (mg/L)^a^	89.1 ± 64.4	123.1 ± 66.9	67.7 ± 52.5	<0.001
Postoperative CAR^a^	2.8 ± 2.3	4.2 ± 2.6	2.0 ± 1.6	<0.001
Surgical indication, *n* (%)^c^				<0.001
Malignant	299 (83.3)	97 (69.8)	202 (91.8)	
Non-malignant	60 (16.7)	42 (30.2)	18 (8.2)	
ASA ≥3, *n* (%)^c^	27 (7.5)	16 (11.5)	11 (5.0)	0.023
Intraoperative blood transfusion, *n* (%)^c^	19 (5.3)	11 (7.9)	8 (3.6)	0.078

*CRP* C-reactive protein, *ALB* albumin, *POD* postoperative day, *CAR* CRP to albumin (CRP/ALB) ratio

^a^Values are expressed as the mean ± SD

^b^Values are expressed as the median (range)

^c^Values are expressed as *n* (%)

### Analysis of possible risk factors for postoperative complications

As shown in Table 1, univariate analysis revealed factors that were significantly associated with postoperative complications, including sex, BMI, preoperative hemoglobin, estimated blood loss, stoma creation, CRP on POD 3, postoperative CAR, surgical indication, and ASA. Then, a multivariate analysis model was used to determine the risk factors from the univariate analysis that were independently associated with postoperative complications. In the multivariate analysis, postoperative CAR (OR 4.413; 95% CI 2.463–7.906; *P* < 0.001) was found to be the only significant independent risk factor for postoperative complications (Table 2). However, we cannot draw conclusions solely from this result that postoperative CAR can act as an accurate predictor for postoperative complications.

**Table 2:** Multivariate analysis of factors associated with postoperative complications

Risk factor	OR	95% CI	*P* value
Male sex	1.307	0.738–2.313	0.359
BMI (<18.5 kg/m^2^)	0.928	0.389–2.216	0.867
Preoperative hemoglobin (<120 g/L)	1.805	1.053–3.093	0.032
Estimated blood loss	1.002	1.000–1.004	0.021
Stoma creation	1.095	0.542–2.209	0.801
CRP on POD 3 (>135 mg/L)	1.826	0.938–3.556	0.077
Postoperative CAR (>2.2)	4.413	2.463–7.906	<0.001
Surgical indication	0.466	0.194–1.116	0.087
ASA ≥3	1.380	0.525–3.624	0.514

*CRP* C-reactive protein, *ALB* albumin, *POD* postoperative day, *CAR* CRP to albumin (CRP/ALB) ratio

### Predictive accuracy of postoperative CAR and CRP on POD 3 for postoperative complications

**Fig. 1:**
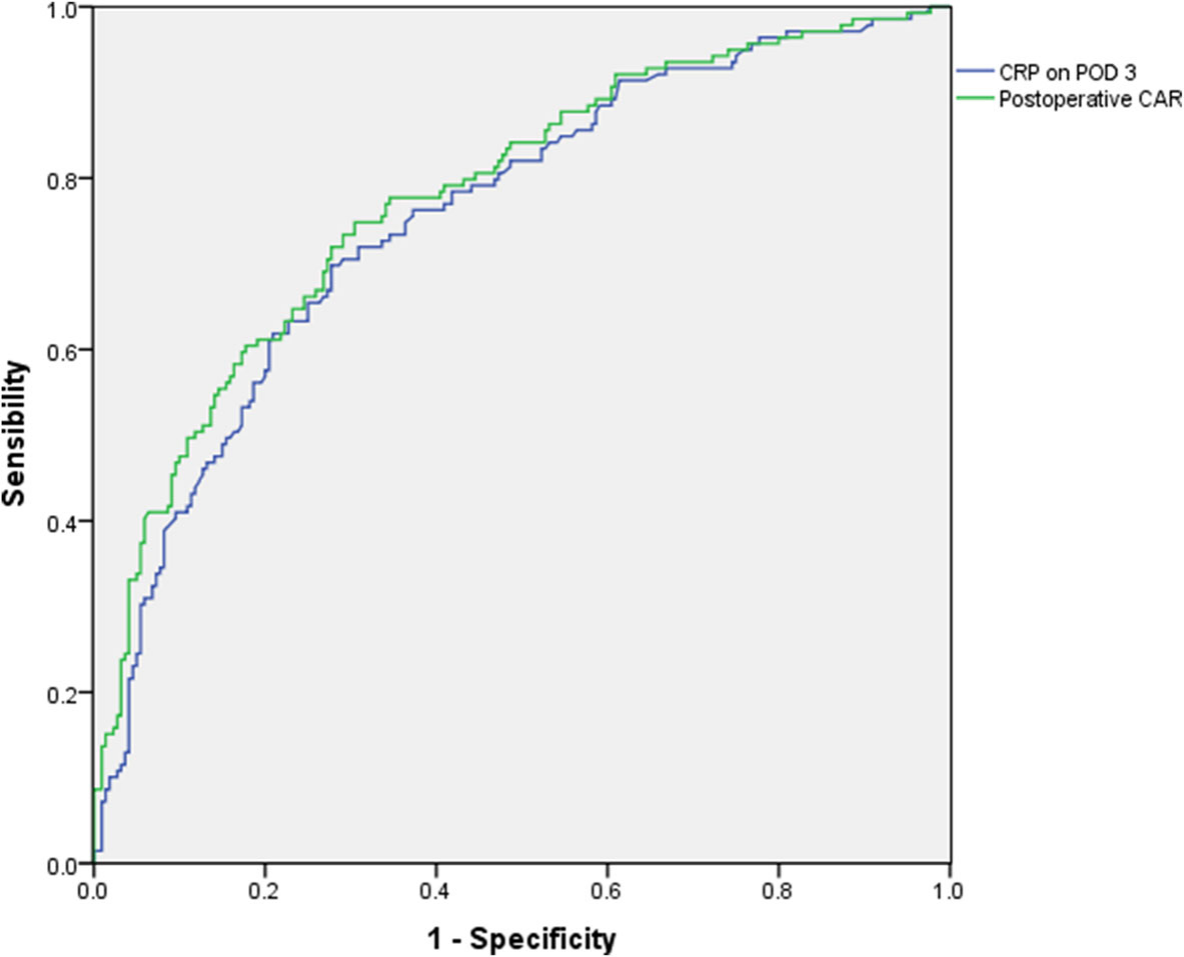
ROC curve showing postoperative CAR levels and CRP on POD 3 levels predictive of postoperative overall complications

Many studies have already suggested that CRP on POD 3 or 4 could be a practical predictive index for postoperative complications in colorectal surgery [1]. ROC curve analysis was used to examine the predictive accuracy of postoperative CAR vs CRP on POD 3. The ROC curve parameters are shown in Table 3 and Fig. 1. For CRP on POD 3, the area under the curve (AUC) was 0.756, sensitivity was 0.698, specificity was 0.723, positive predictive value was 79.1%, negative predictive value was 61.4%, Youden’s index was 0.421, and the cutoff point was 85.1. On the other hand, the AUC of CAR was 0.779, sensitivity was 0.748, specificity was 0.695, positive predictive value was 81.4%, negative predictive value was 60.8%, Youden’s index was 0.444, and the cutoff point was 2.2. Clinically, the positive predictive value (PPV) is used as a practical predictive parameter. For example, the PPV of CRP was 79.1%, and the PPV of CAR was 81.4%, which means the probability of correctly predicting complications in a patient with CRP ≥ 85.1 was 79.1%; however, the probability was 81.4% in a patient with CAR ≥2.2. Therefore, CAR was a better predictor than CRP on POD 3 in patients undergoing colorectal surgery.

**Table 3:** ROC curve showing Postoperative CAR levels and CRP on POD 3 levels predictive of postoperative overall complications

	Postoperative CAR	CRP on POD 3
Cutoff point	2.2	85.1
AUC	0.779	0.756
Sensitivity	0.748	0.698
Specificity	0.695	0.723
Positive predictive value	81.4%	79.1%
Negative predictive value	60.8%	61.4%
Youden’s index	0.444	0.421

*CAR* CRP to albumin (CRP/ALB) ratio, *ROC* receiver operating characteristic, *CRP* C-reactive protein, *ALB* albumin, *POD* postoperative day

### Use of CAR to predict postoperative complications

Patients were separated into two groups based on the cutoff values. Patients with CAR ≥2.2 were found to have more postoperative complications than those with CAR <2.2 (60.8% vs 18.6%, *P* < 0.001). Patients with CAR ≥2.2 had more mild complications (grade I to II) and major complications than those with CAR <2.2 (39.8% vs 14.9%, *P* < 0.001; 25.1% vs 6.4%, *P* < 0.001, respectively). Moreover, patients with CAR ≥2.2 were more likely to have a longer postoperative stay and surgical site infection (*P* < 0.001) (Table 4).

**Table 4:** Comparison of postoperative complications associated with postoperative CAR

Characteristics	CAR <2.2 (*n* = 188)	CAR ≥2.2 (*n* = 171)	*P* value
Overall, *n* (%)^a, c^	35 (18.6)	104 (60.8)	<0.001
Grade I, *n* (%)^a, c^	12 (6.4)	25 (14.6)	0.010
Ileus	4 (2.1)	5 (2.9)	
Fever >38.5 °C after surgery	5 (2.7)	10 (5.8)	
Delayed gastric emptying	1 (0.5)	3 (1.8)	
Wound infection	2 (1.1)	5 (2.9)	
Urinary retention	0	1 (0.6)	
Transient confusion	0	1 (0.6)	
Grade II, *n* (%)^a, c^	16 (8.5)	43 (25.1)	<0.001
Postoperative blood transfusions >2U	9 (4.8)	25 (14.6)	
TPN >2 weeks	3 (1.6)	5 (2.9)	
Pneumonia	2 (1.1)	2 (1.2)	
Urinary tract infection	0	1 (0.6)	
Wound infection	0	7 (4.1)	
Early postoperative bowel obstruction	2 (1.1)	0	
Line sepsis	0	3 (1.8)	
Grade III, *n* (%)^a, c^	11 (5.9)	38 (22.2)	<0.001
Fascial dehiscence	2 (1.1)	2 (1.2)	
Abdomino-pelvic collection	2 (1.1)	3 (1.8)	
Pleural effusion	1 (0.5)	7 (4.1)	
Gastrointestinal bleeding	0	1 (0.6)	
Acalculous choleycystitis	0	3 (1.8)	
Intraabdominal bleeding	0	1 (0.6)	
Anastomotic leakage	3 (1.6)	15 (8.8)	
Lymphatic leakage	1 (0.5)	0	
Stoma complications	0	1 (0.6)	
Early postoperative bowel obstruction	0	1 (0.6)	
Intraabdominal abscess	2 (1.1)	4 (2.3)	
Grade IV, *n* (%)^a, c^	1 (0.5)	5 (2.9)	0.176
Septic shock	0	1 (0.6)	
Sepsis	0	1 (0.6)	
Respiratory failure	0	1 (0.6)	
Kidney failure	0	1 (0.6)	
MODS	1 (0.5)	1 (0.6)	
Grade V, *n* (%)^a, c^	0	0	
Grade III or greater, *n* (%)^a, c^	12 (6.4)	43 (25.1)	<0.001
Postoperative stay (days)^b^	7 (3–78)	10 (4–71)	<0.001
Surgical site infection (+), SSI (+), *n* (%)^c^	9 (4.8)	36 (21.1)	<0.001

*CAR* CRP to albumin (CRP/ALB) ratio, *CRP* C-reactive protein, *ALB* albumin

^a^Clavien-Dindo’s classification of surgical complication

^b^Values are expressed as the median (range)

^c^Values are expressed as *n* (%)

### Predictive value of CAR in patients with colorectal cancer

A subgroup analysis of the predictive value of CAR in patients with colorectal cancer was performed. Receiver operating characteristic curve analysis showed that the AUC of CAR in patients with colorectal cancer was 0.764, with a sensitivity of 0.691 and a specificity of 0.728. The cutoff value was 2.3. The AUC of CRP on POD 3 was 0.747. The sensitivity was 0.670, and the specificity was 0.743. The cutoff value was 85.1. Univariate and multivariate analyses showed that CAR ≥2.3 (OR 4.152, 95% CI 2.233–7.721, *P* < 0.001) was an independent risk factor for postoperative morbidity. Patients with colorectal cancer with CAR ≥2.3 were more likely to have longer postoperative hospital stays (*P* < 0.001), more SSIs (*P* < 0.001), and more postoperative complications (*P* < 0.001).

## Discussion

The main findings of our study can be summarized as follows. First, CAR was an independent and significant risk factor for postoperative complications in patients undergoing colorectal surgery. Second, CAR appeared to show higher accuracy than CRP on POD 3 alone as a predictor of postoperative complications. Third, patients with CAR ≥2.2, which was more accurate than CRP level on POD 3, were at higher risk of postoperative complications, prolonged postoperative hospital stay and more SSIs after colorectal surgery.

Increasing evidence shows that a systemic inflammatory response following surgical trauma is associated with poor outcome in patients after surgery, and this was revealed by serum C-reactive protein and albumin [9, 16, 17]. There are some inflammation-based prognostic scores, such as the modified Glasgow Prognostic Score (mGPS), neutrophil-to-lymphocyte ratio (NLR), platelet-to-lymphocyte ratio (PLR), postoperative Glasgow Prognostic Score (poGPS), and CRP to albumin ratio (CAR), that predict outcomes for surgical patients [15, 18–20]. Watt et al. [20] suggested that a postoperative systemic inflammation score including postoperative C-reactive protein and albumin levels could predict short- and long-term outcomes in patients undergoing colorectal surgery. Among these, CAR is novel. Haruki et al. [19] suggested that for pancreatic cancer patients after pancreatic resection, CAR could be an independent and significant indicator of poor long-term outcome. Shibutani et al. [21] found preoperative CAR to be a useful prognostic marker in patients with colorectal cancer undergoing potentially curative surgery. In septic patients, Ranzani et al. [15] also reported that residual inflammation at ICU discharge as assessed by CAR was an independent risk factor for outcome.

It is well known that postoperative CRP levels can be used as a practical marker to assess postoperative inflammation and to predict postoperative complications [1]. However, some studies still find certain drawbacks to CRP as a predictor of stress-related complications [9, 22], including less accuracy in some patients. However, CAR is based on circulating levels of two acute phase proteins, CRP and albumin, which are also associated with inflammation from surgical trauma [19]. In our study, CAR was an independent prognostic factor for postoperative complications in multivariate analysis (OR 4.413; 95% CI 2.463–7.906; *P* < 0.001). In contrast, CRP on POD 3 was not an independent risk factor for complications after colorectal surgery in multivariate analysis (OR 1.826; 95% CI 0.938–3.556; *P* = 0.077). ROC analysis also revealed that the AUC of CAR was higher than that of CRP, and CAR had a higher positive predictive value than CRP on POD 3. Combined with ROC analysis, these results suggest that the predictive value of CAR was superior to that of CRP on POD 3, and CAR appeared to show higher accuracy than CRP alone.

The reasons why CAR could be more accurate than CRP alone for predicting postoperative complications might be that CRP combined with albumin is more likely to reflect inflammation from surgical stress. Studies have already proven that the surgical stress response can be evidenced by CRP concentrations on POD 3 or 4 [1, 7, 23]. Moreover, serum albumin acting as a negative acute-phase protein also decreases in response to surgical stress [24]. Increased capillary leakage of albumin is one of the features of the systemic inflammatory response after colorectal surgery, which leads to changes in serum albumin levels [16]. In addition, collagen synthesis and granuloma formation, which impairs the innate immune response, will be influenced by hypoalbuminemia; therefore, wound healing is delayed, and the systemic immune status is predisposing to infection [25]. Although many studies have focused on serum albumin before surgery, Hubner et al. [9] showed an early postoperative albumin drop to be related to adverse clinical outcomes. Lee et al. [26] found that after oral cancer surgery, patients with postoperative hypoalbuminemia were at risk of surgical site infection. Hypoalbuminemia is thought to be associated with inflammation or previous malnutrition [27]. Some studies have already merged albumin and CRP into a single index to predict outcomes [12, 28]. In our study, we found that an increased CRP/albumin ratio could reflect higher diagnostic accuracy for postoperative complications than CRP alone. As a result, it is better to put albumin and CRP together to predict postoperative complications.

The current study had several limitations. First, it is a retrospective observational analysis, and the effects of residual confounding factors cannot be excluded completely. Second, it is a single-center study, and it is necessary to conduct multicenter studies for further confirmation, as perioperative management is dependent on our local experience. Third, other surgeries, such as gastrectomy, esophagectomy resection, and liver resection, are needed to verify these results.

## Conclusions

The current study confirmed that the CRP/albumin ratio predicted postoperative outcomes in patients who underwent elective colorectal surgery. Merging CRP and albumin into a single index could be more accurate than CRP alone. Patients with CAR greater than 2.2 should be intensively monitored for early detection of postoperative outcome to reduce postoperative complications, shorten hospital stay, quickly return to premorbid functional activity, and reduce hospital cost.

## Abbreviations

ALB: Albumin
ASA: American Society of Anesthesiologists
CRP: C-reactive protein
POD: Postoperative day
ROC: Receiver operating characteristic
SIRS: Systemic inflammatory response syndrome

## References

[1] Warschkow R, Beutner U, Steffen T, Muller SA, Schmied BM, Guller U (2012). Safe and early discharge after colorectal surgery due to C-reactive protein: a diagnostic meta-analysis of 1832 patients. Ann Surg.

[2] Zhang T, Cao L, Cao T, Yang J, Gong J, Zhu W (2015). Prevalence of sarcopenia and its impact on postoperative outcome in patients with Crohn’s disease undergoing bowel resection. JPEN J Parenter Enteral Nutr.

[3] Frolkis AD, Dykeman J, Negron ME, Debruyn J, Jette N, Fiest KM (2013). Risk of surgery for inflammatory bowel diseases has decreased over time: a systematic review and meta-analysis of population-based studies. Gastroenterology.

[4] Krarup PM, Nordholm-Carstensen A, Jorgensen LN, Harling H (2014). Anastomotic leak increases distant recurrence and long-term mortality after curative resection for colonic cancer: a nationwide cohort study. Ann Surg.

[5] Ortega-Deballon P, Radais F, Facy O, d'Athis P, Masson D, Charles PE (2010). C-reactive protein is an early predictor of septic complications after elective colorectal surgery. World J Surg.

[6] Watt DG, McSorley ST, Horgan PG, McMillan DC (2015). Enhanced recovery after surgery: which components, if any, impact on the systemic inflammatory response following colorectal surgery?: a systematic review. Medicine.

[7] Facy O, Paquette B, Orry D, Binquet C, Masson D, Bouvier A (2016). Diagnostic accuracy of inflammatory markers as early predictors of infection after elective colorectal surgery: results from the IMACORS study. Ann Surg.

[8] Norberg A, Rooyackers O, Segersvard R, Wernerman J (2015). Albumin kinetics in patients undergoing major abdominal surgery. PLoS One.

[9] Hubner M, Mantziari S, Demartines N, Pralong F, Coti-Bertrand P, Schafer M (2016). Postoperative albumin drop is a marker for surgical stress and a predictor for clinical outcome: a pilot study. Gastroenterol Res Pract.

[10] Lee EH, Chin JH, Choi DK, Hwang BY, Choo SJ, Song JG (2011). Postoperative hypoalbuminemia is associated with outcome in patients undergoing off-pump coronary artery bypass graft surgery. J Cardiothorac Vasc Anesth.

[11] Ishizuka M, Nagata H, Takagi K, Iwasaki Y, Shibuya N, Kubota K (2016). Clinical significance of the C-reactive protein to albumin ratio for survival after surgery for colorectal cancer. Ann Surg Oncol.

[12] Fairclough E, Cairns E, Hamilton J, Kelly C (2009). Evaluation of a modified early warning system for acute medical admissions and comparison with C-reactive protein/albumin ratio as a predictor of patient outcome. Clin Med.

[13] Sang BH, Bang JY, Song JG, Hwang GS (2015). Hypoalbuminemia within two postoperative days is an independent risk factor for acute kidney injury following living donor liver transplantation: a propensity score analysis of 998 consecutive patients. Crit Care Med.

[14] Dindo D, Demartines N, Clavien PA (2004). Classification of surgical complications: a new proposal with evaluation in a cohort of 6336 patients and results of a survey. Ann Surg.

[15] Ranzani OT, Zampieri FG, Forte DN, Azevedo LC, Park M (2013). C-reactive protein/albumin ratio predicts 90-day mortality of septic patients. PLoS One.

[16] Fleck A, Raines G, Hawker F, Trotter J, Wallace PI, Ledingham IM (1985). Increased vascular permeability: a major cause of hypoalbuminaemia in disease and injury. Lancet.

[17] Angiolini MR, Gavazzi F, Ridolfi C, Moro M, Morelli P, Montorsi M (2016). Role of C-reactive protein assessment as early predictor of surgical site infections development after pancreaticoduodenectomy. Dig Surg.

[18] Proctor MJ, Morrison DS, Talwar D, Balmer SM, Fletcher CD, O'Reilly DS (2011). A comparison of inflammation-based prognostic scores in patients with cancer. A Glasgow Inflammation Outcome Study. Eur J Cancer.

[19] Haruki K, Shiba H, Shirai Y, Horiuchi T, Iwase R, Fujiwara Y (2016). The C-reactive protein to albumin ratio predicts long-term outcomes in patients with pancreatic cancer after pancreatic resection. World J Surg.

[20] Watt DG, McSorley ST, Park JH (2016). A postoperative systemic inflammation score predicts short- and long-term outcomes in patients undergoing surgery for colorectal cancer. Ann Surg Oncol.

[21] Shibutani M, Maeda K, Nagahara H, Iseki Y, Ikeya T, Hirakawa K (2016). Prognostic significance of the preoperative ratio of C-reactive protein to albumin in patients with colorectal cancer. Anticancer Res.

[22] Easton R, Balogh ZJ (2014). Peri-operative changes in serum immune markers after trauma: a systematic review. Injury.

[23] Korner H, Nielsen HJ, Soreide JA, Nedrebo BS, Soreide K, Knapp JC (2009). Diagnostic accuracy of C-reactive protein for intraabdominal infections after colorectal resections. J Gastrointest Surg.

[24] Sung J, Bochicchio GV, Joshi M, Bochicchio K, Costas A, Tracy K (2004). Admission serum albumin is predicitve of outcome in critically ill trauma patients. Am Surg.

[25] Otranto M, Souza-Netto I, Aguila MB, Monte-Alto-Costa A (2009). Male and female rats with severe protein restriction present delayed wound healing. Appl Physiol Nutr Metab.

[26] Lee JI, Kwon M, Roh JL, Choi JW, Choi SH, Nam SY (2015). Postoperative hypoalbuminemia as a risk factor for surgical site infection after oral cancer surgery. Oral Dis.

[27] Vincent JL, Dubois MJ, Navickis RJ, Wilkes MM (2003). Hypoalbuminemia in acute illness: is there a rationale for intervention? A meta-analysis of cohort studies and controlled trials. Ann Surg.

[28] Pinilla JC, Hayes P, Laverty W, Arnold C, Laxdal V (1998). The C-reactive protein to prealbumin ratio correlates with the severity of multiple organ dysfunction. Surgery.

